# Supplementary figures and images for: Discovery of non-retinoid compounds that suppress the pathogenic effects of misfolded rhodopsin in a mouse model of retinitis pigmentosa
Source: PLoS Biol. 2025 Jan 14;23(1):e3002932. doi: 10.1371/journal.pbio.3002932 (PMC11731721; doi:10.1371/journal.pbio.3002932)

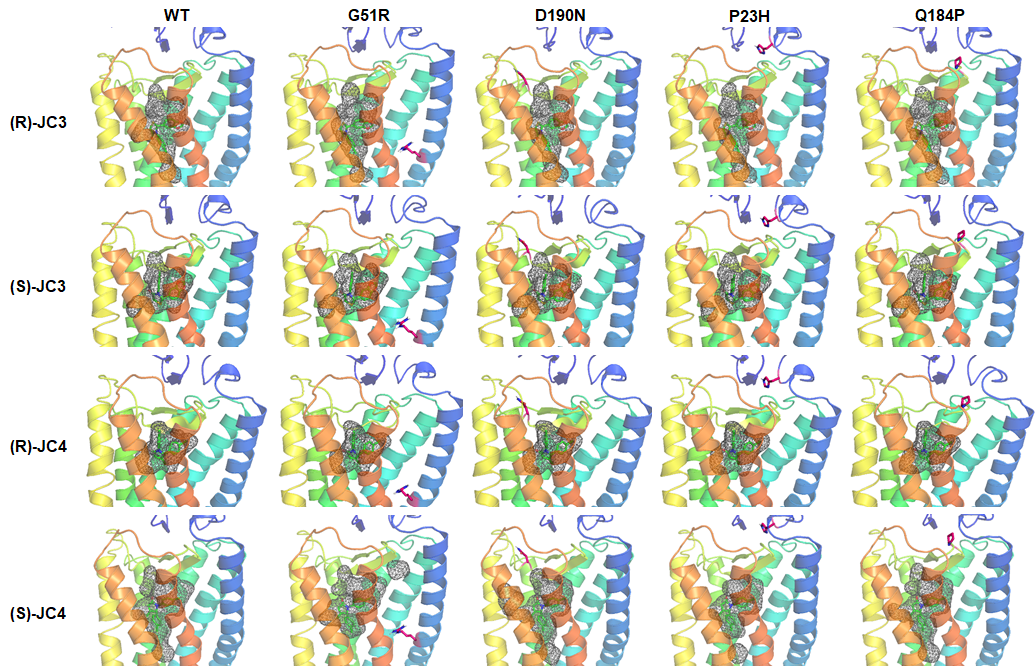

Supplement: S4 Fig — Binding pockets of JC3- and JC4-bound human rod opsin homology model and variants. The mutated amino acids are shown in magenta. (TIF) [file pbio.3002932.s004.tif]

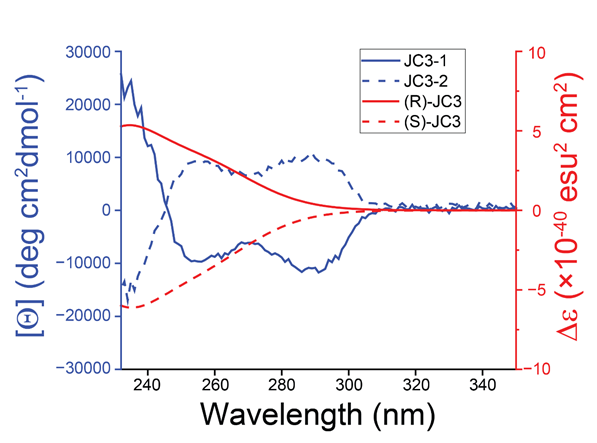

Supplement: S5 Fig — The isolated enantiomers of JC3 were assigned by the comparison of computed and measured circular dichroism spectra. The CD spectra of isomer 1 (solid blue) and isomer 2 (dashed blue) were recorded in acetonitrile, and their molar ellipticities were plotted against the wavelength. For the sake of comparison, the electronic structures of the R (solid red) and S (dashed red) enantiomers of JC3 were modeled using density functional theory in order to simulate their CD spectra. The computed change in ellipticity for each enantiomer is plotted against the wavelength for the sake of comparison for the lowest energy conformer, which corresponds to the major peak in each experimental structure. The coincidence between the major peaks suggests isomer 1 is R and isomer 2 is S. The numerical data can be found in S1 Data. (TIF) [file pbio.3002932.s005.tif]

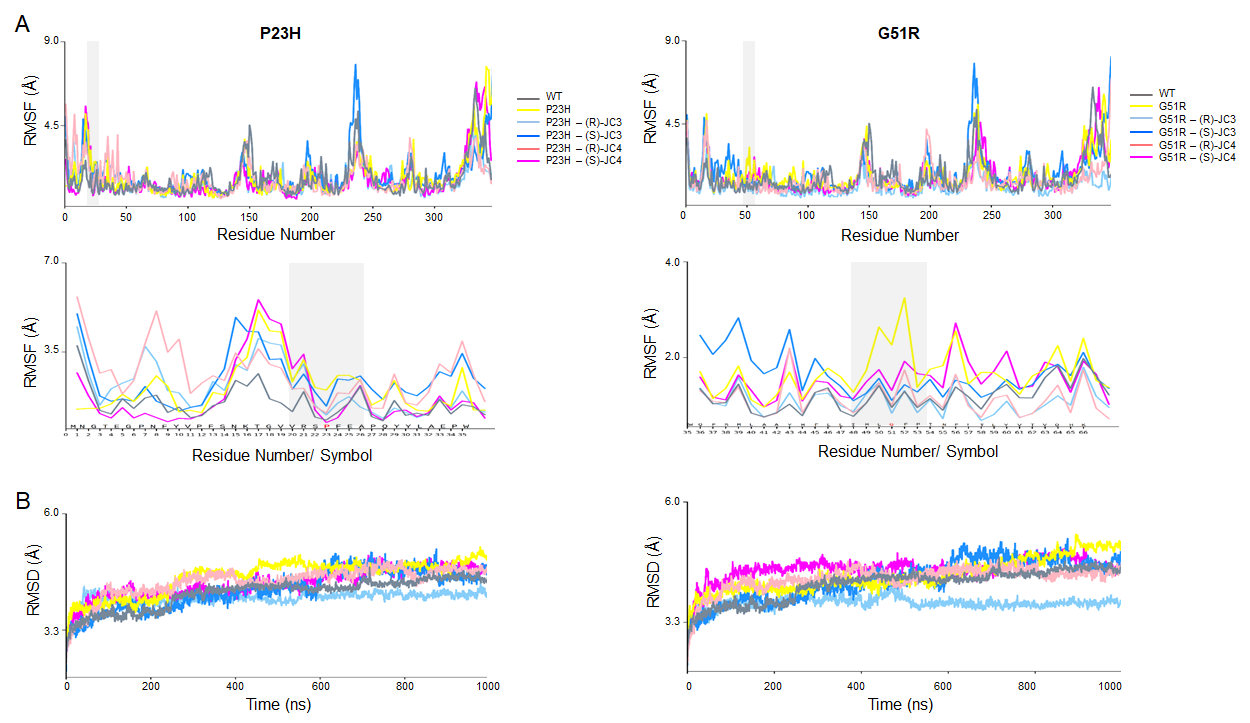

Supplement: S7 Fig — The molecular dynamic (MD) simulations were carried out for the JC3- and JC4-bound human rod opsin homology model and variants. (A) The root mean square fluctuation (RMSF). (B) The root mean square deviation (RMSD). The numerical data can be found in S1 Data. (TIF) [file pbio.3002932.s007.tif]

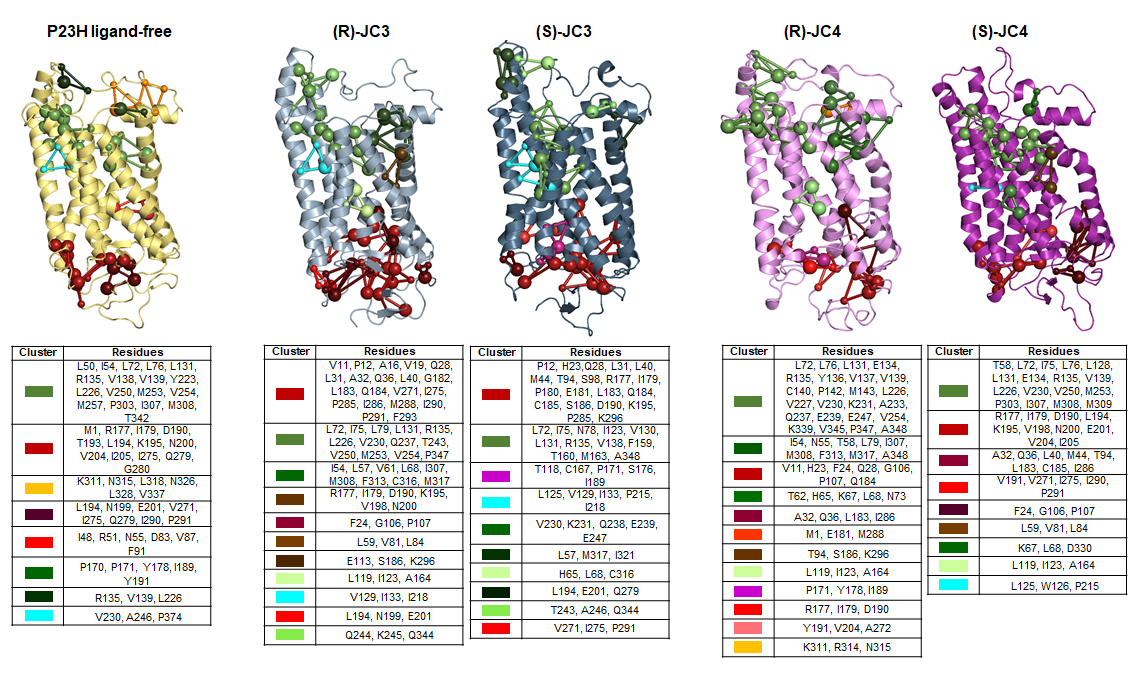

Supplement: S8 Fig — The specific residue interaction clusters are shown in the protein structures and specified in the tables. A PSN analysis [33] carried out based on MD simulations identified clusters of stabilizing interactions within the native rod opsin structure. The binding of either JC3 or JC4 increased the number of interacting residues within the native clusters or generated additional clusters of interacting residues. Thus, JC compounds generally appear to stabilize the native fold by improving internal residue–residue interactions that form in the context of the RP variants. (TIF) [file pbio.3002932.s008.tif]

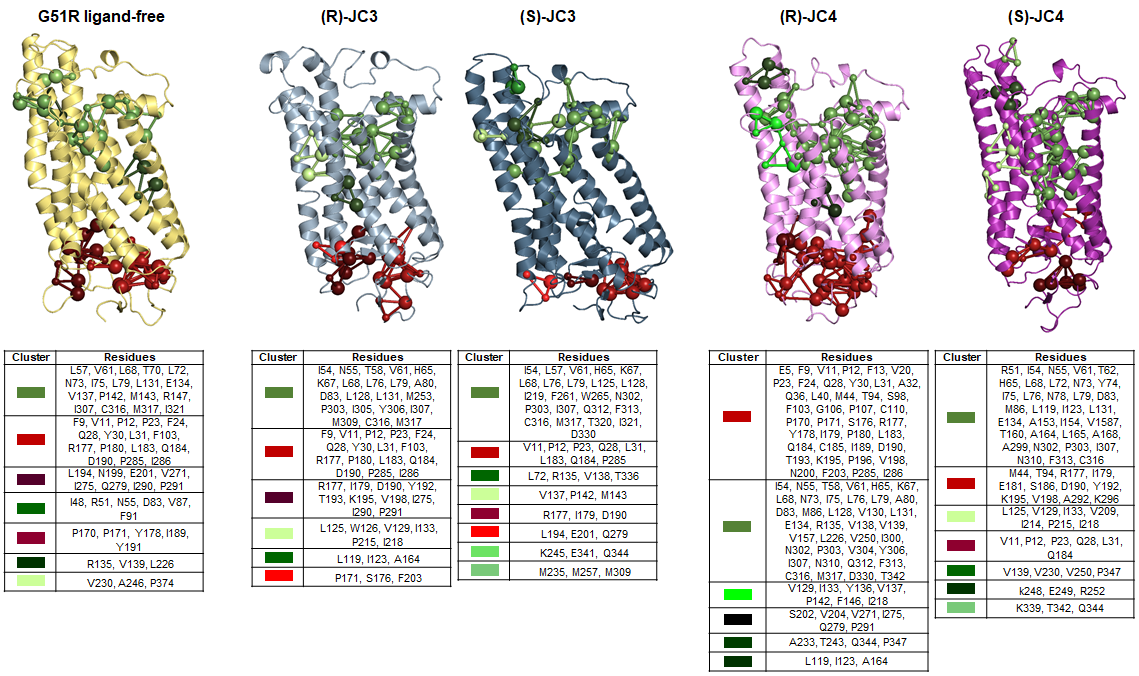

Supplement: S9 Fig — The specific residue interaction clusters are shown in the protein structures and specified in the tables. A PSN analysis [33] carried out based on MD simulations identified clusters of stabilizing interactions within the native rod opsin structure. The binding of either JC3 or JC4 increased the number of interacting residues within the native clusters or generated additional clusters of interacting residues. Thus, JC compounds generally appear to stabilize the native fold by improving internal residue–residue interactions that form in the context of the RP variants. (TIF) [file pbio.3002932.s009.tif]
