# Supplementary material for: Discovery of non-retinoid compounds that suppress the pathogenic effects of misfolded rhodopsin in a mouse model of retinitis pigmentosa
Source: PLoS Biol. 2025 Jan 14;23(1):e3002932. doi: 10.1371/journal.pbio.3002932 (PMC11731721; doi:10.1371/journal.pbio.3002932)
Supplement: S1 Raw Images — (PPTX) [file pbio.3002932.s010.pptx]

## Slide 1
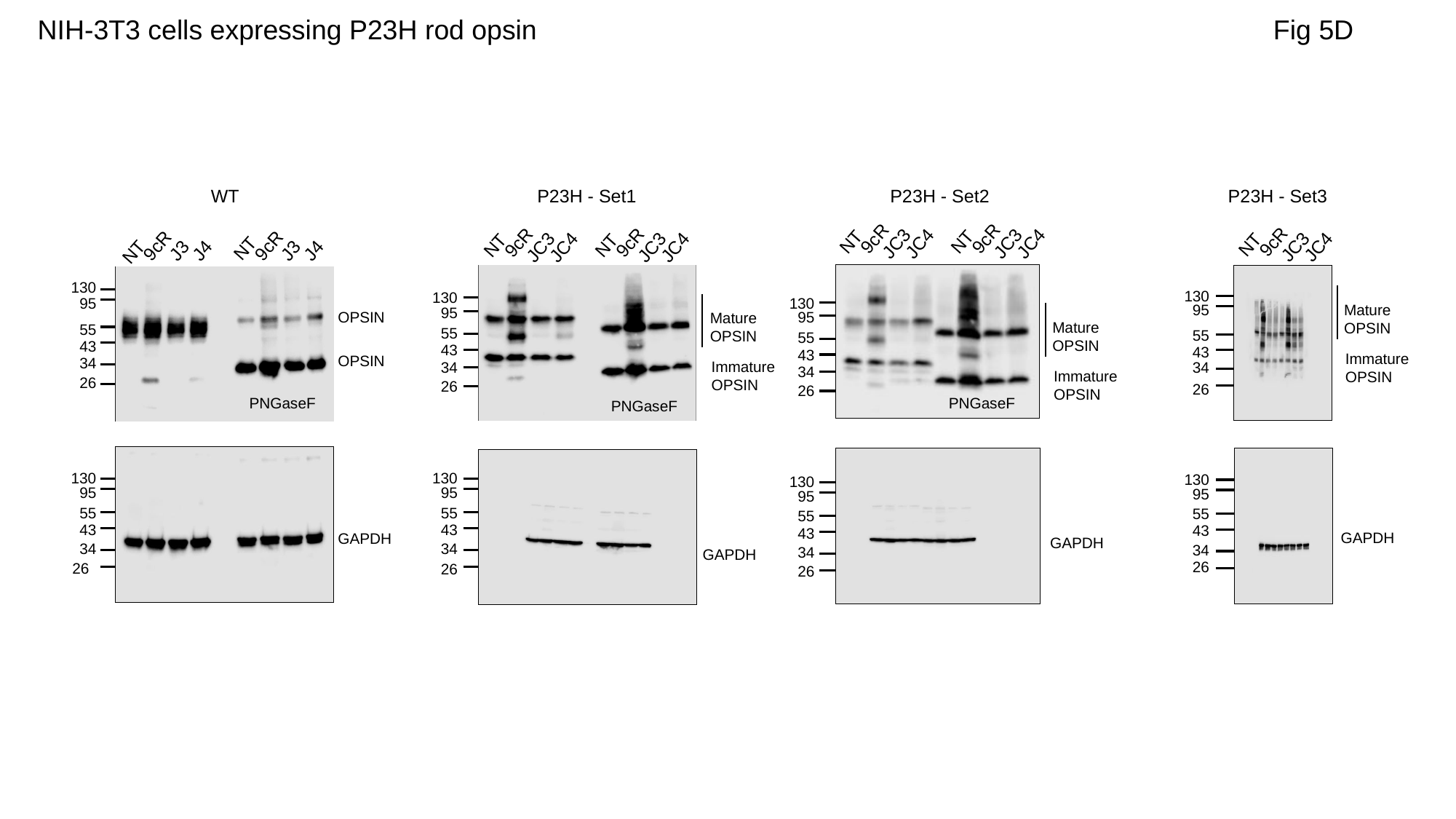

NIH-3T3 cells expressing P23H rod opsin
Fig 5D
WT
P23H - Set1
P23H - Set2
P23H - Set3
9cR
9cR
NT
NT
9cR
9cR
9cR
JC3
JC4
JC3
JC4
NT
NT
NT
9cR
9cR
JC3
JC4
JC3
JC4
JC3
JC4
NT
J3
J4
J3
J4
NT
130
130
130
130
95
Mature
OPSIN
95
95
OPSIN
95
Mature
OPSIN
Mature
OPSIN
55
55
55
55
43
43
43
43
Immature
OPSIN
OPSIN
34
Immature
OPSIN
34
34
34
Immature
OPSIN
26
26
26
26
PNGaseF
PNGaseF
PNGaseF
130
130
130
130
95
95
95
95
55
55
55
55
43
43
43
43
GAPDH
GAPDH
GAPDH
34
34
34
34
GAPDH
26
26
26
26

## Slide 2
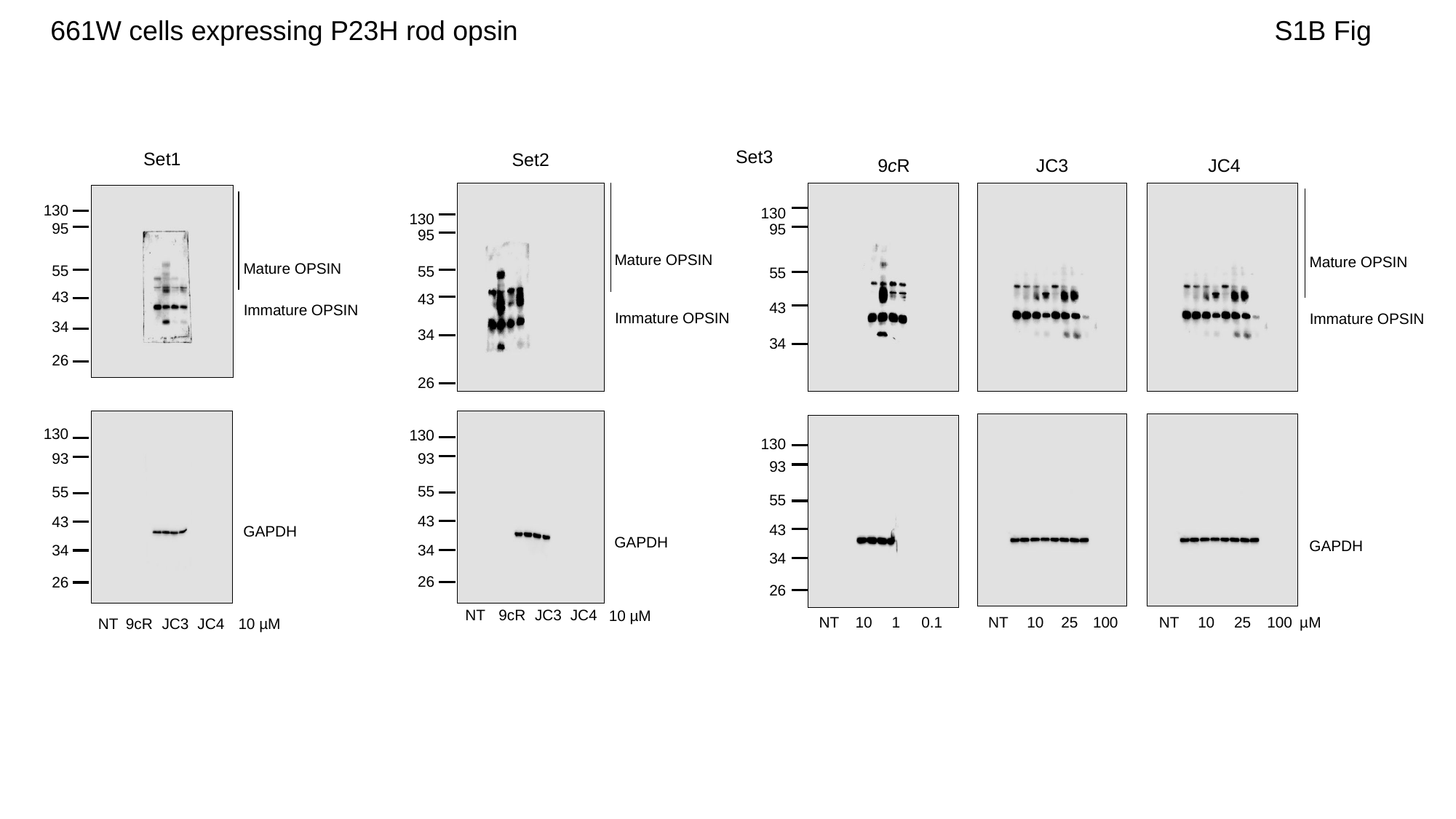

661W cells expressing P23H rod opsin
S1B Fig
Set3
Set1
Set2
9cR
JC3
JC4
130
130
130
95
95
95
Mature OPSIN
Mature OPSIN
Mature OPSIN
55
55
55
43
43
43
Immature OPSIN
Immature OPSIN
Immature OPSIN
34
34
34
26
26
130
130
130
93
93
93
55
55
55
43
43
43
GAPDH
GAPDH
GAPDH
34
34
34
26
26
26
NT
9cR
JC3
JC4
10 µM
NT
10
1
0.1
NT
10
25
100
NT
10
25
100
µM
JC4
10 µM
9cR
JC3
NT

## Slide 3
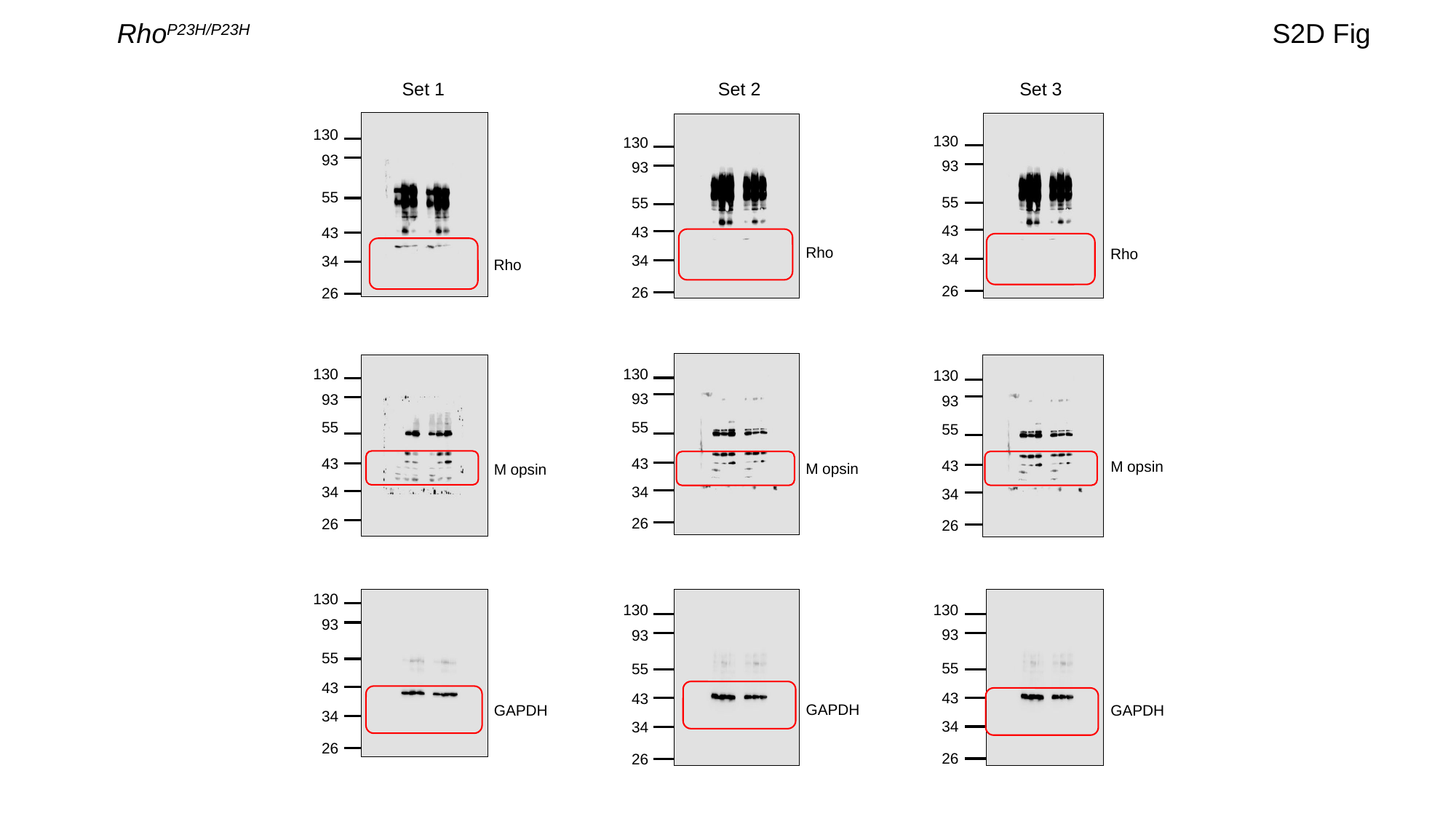

RhoP23H/P23H
S2D Fig
Set 1
Set 2
Set 3
130
130
130
93
93
93
55
55
55
43
43
43
Rho
Rho
34
34
34
Rho
26
26
26
130
130
130
93
93
93
55
55
55
43
43
43
M opsin
M opsin
M opsin
34
34
34
26
26
26
130
130
130
93
93
93
55
55
55
43
GAPDH
43
43
GAPDH
GAPDH
GAPDH
34
34
34
26
26
26
